# The Long-Term Effects of Financial Hardship on Health: Pre and Post the Great Recession

**DOI:** 10.1093/geroni/igaa057.2432

**Published:** 2020-12-16

**Authors:** Gillian Marshall

**Affiliations:** University of Washington, Seattle, Washington, United States

## Abstract

Purpose: Now 10 years after the Great Recession of 2018, we examined the impact and long-term health outcomes impacting adults (≥ 50 years). Methods: Data from the Health and Retirement Survey (sample n=5,160), was used to examine how changes in financial hardship pre-post the Great Recession of 2008 impacted the likelihood of developing new chronic conditions in 2016. Results: Preliminary results suggest that reduced medication use during the recession was significantly correlated with a higher likelihood of developing arthritis, lung disease, psychological conditions, depression, and greater deterioration of mental and physical health relative to an absence of reduced medication use in 2006 and 2010 (1). Conclusion: These findings underscore the adverse influences of increased financial hardships that impact medication use during recessionary periods on long term health and wellbeing of older adults. They also provide evidence of deleterious effects on health of difficulty paying bills throughout the study period.

